# Physical, Mechanical and Radiological Characteristics of a Fly Ash Geopolymer Incorporating Titanium Dioxide Waste as Passive Fire Insulating Material in Steel Structures

**DOI:** 10.3390/ma15238493

**Published:** 2022-11-29

**Authors:** Pedro Antonio Salazar, Carlos Leiva Fernández, Yolanda Luna-Galiano, Rosario Villegas Sánchez, Constantino Fernández-Pereira

**Affiliations:** Chemical and Environmental Engineering Department, School of Engineering, University of Seville, Camino de los Descubrimientos s/n 41092, 41092 Seville, Spain

**Keywords:** fire resistance, fly ashes, titanium dioxide waste, mechanical properties, radionuclide

## Abstract

This research analyzes whether a titanium dioxide waste (TiO_2_ waste) can be used as a source material for geopolymers with good fire resistance properties. Samples with different proportions were prepared, replacing fly ashes with titanium dioxide waste on geopolymers (0, 20, 30, 40 and 100% *w*/*w*). The activating solution has a Na_2_O/SiO_2_ molar ratio of 0.98. Physical (bulk density, moisture content and water absorption) and mechanical (superficial hardness and compressive strength) characteristics have been evaluated. In addition, their thermal behavior at high temperatures (fire resistance, compressive strength at elevated temperature and absorbed energy) has also been evaluated to see if they can be used as fire insulating materials. This work also studies the radiological activity of geopolymer materials. The replacement of FA with WTiO_2_ increases the bulk density due to its higher specific bulk density. The highest compressive strength values were obtained with a TiO_2_ waste content between 30 and 40% *w*/*w*. The compressive strength decreases at high temperatures, especially when more TiO_2_ waste is added. When the amount of TiO_2_ waste is increased, so is the plateau of evaporation, and this, in turn, increases the resistance to fire. Geopolymers containing FA and TiO_2_ waste do not present radiological problems, although, when the TiO_2_ waste is increased, the activity index of the geopolymer also rises.

## 1. Introduction

The development of new building materials has advanced dramatically in recent years. In the 1980s, Professor Joseph Davidovits named inorganic polymers of aluminosilicates synthesized by geopolymerization reactions “geopolymers”. They are produced as a consequence of the chemical reaction between a solid aluminosilicate and an aqueous solution of alkali silicates or hydroxides, at room or slightly higher temperatures, to give a new synthetic alkali aluminosilicate [1]. Geopolymer design has opened a new field of research, since they can provide behaviors comparable to those of other cementitious materials.

Geopolymers are characterized by their high mechanical strength, fire resistance, resistance to acids, low thermal conductivity and fast setting times, depending on the raw materials used [2,3] and manufacturing conditions [4,5]. Although not all geopolymers present all the properties mentioned, the knowledge currently obtained allows formulations to be defined under the appropriate conditions to obtain the ideal properties for a specific application [6]. 

The term geopolymer is also used to refer to inorganic polymeric concretes [7,8] or cements activated by alkaline routes [9]. In some cases, the differences between them are due to their properties and characteristics of the aluminosilicates used as precursors or the presence or absence of soluble silicates and/or alkaline hydroxides [10]. In this work, this term has always been used to refer to the cementitious material obtained as a result of the alkaline activation of a silica–aluminous-type precursor. Coal combustion fly ashes have been used widely as precursors in geopolymers [11] due to the potentially active silica–alumina content. However, from 2018 to 2021, the EU reduced the consumption of coal to generate energy by a fourth, according to EuroStat [12], and, thereby, the fly ash production; thus, it is necessary to find new by-products which, when mixed with fly ashes, could improve certain technical requirements of geopolymers in specific applications, such as passive fire protection materials for steel structures.

TiO_2_ waste is produced by digesting ilmenite with concentrated sulfuric acid at temperatures ranging from 150 to 220 °C. Titanium is extracted as titanyl sulfate, which is then hydrolyzed, precipitated and calcined to produce TiO_2_. TiO_2_ waste is usually neutralized or stabilized/solidified before being disposed of in landfills [13]. Diverse metallic impurities, heavy metals and radioisotopes as minor components could make the recycling of building materials difficult without an environmental study [14].

New recycling practices in a variety of processes make this an interesting subject for research. In addition, the proper treatment of industrial waste could lead to the generation of by-products with economic value and wide use, especially when they are recycled for specific uses with high added value which might compete effectively with products derived from traditional raw resources [15]. 

It is important to find ways to protect steel structures from fire as a result of their noninherent fire resistance. Measures to achieve this can be considered active or passive. Active protection includes all actions that have the specific function of detecting and extinguishing the fire once it has already started. Extinguishing methods are based on the elimination of the necessary elements for combustion, such as fuel, oxidant, ignition source or even the possibility of developing chemical chain reactions [16]. Regarding this last item, the main goal of passive protection of steel structures is to delay the time that a steel structure takes to reach the critical temperature at which it loses its mechanical properties, interposing a material that absorbs the heat generated in a fire. This study focuses on the development and evaluation of this latter group of products, adding wastes (TiO_2_ waste) that delay the time of crack structure. Additionally, the influence of different waste doses has been analyzed in the insulating and mechanical properties, as well as in environmental analysis (radiological and leaching) of the materials.

## 2. Materials and Methods

Geopolymer samples have been manufactured using fly ash as a geopolymerization precursor and sodium silicate solution as an activator, incorporating titanium dioxide waste to the matrix. 

### 2.1. Materials

**Figure 1:**
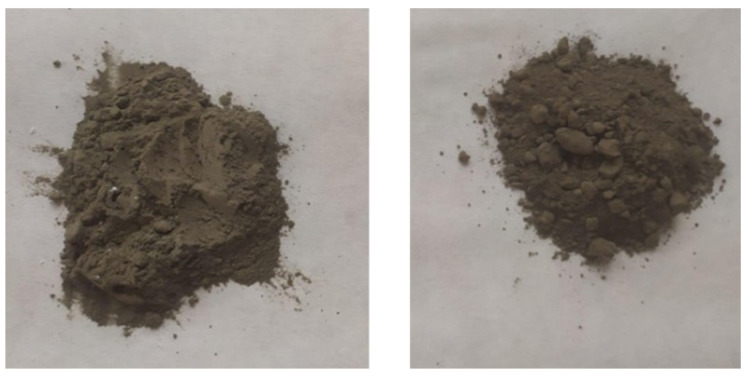
Fly ash (**right**) and titanium dioxide waste (**left**).

Fly ashes (FA) were acquired from a thermal power plant in Los Barrios, Cádiz (Spain) and were obtained from pulverized coal combustion processes at 1500 °C. TiO_2_ waste was obtained after attacking ilmenite with sulfuric acid from a Spanish company. Figure 1 shows what FA and TiO_2_ waste look like. At first glance, differences in their color cannot be seen, as both fly ash and titanium dioxide waste have a similar grayish color.

Main and minor components were determined by means of wavelength dispersive X-ray fluorescence spectrometry. Table 1 and Table 2 show the main chemical and minor components, respectively, of fly ash and titanium dioxide waste.

As observed in Table 1, silicon was the main component of the fly ash. It also had a high content of aluminum and iron. Titanium, silicon and iron were the main components of the titanium dioxide waste, and it presented a high sulfur content in the form of sulfate due to the attack of ilmenite with sulfuric acid [17]. Titanium dioxide waste has a higher specific gravity (2.83) than fly ash (2.17). On the other hand, rubidium was the most abundant minor component in fly ash while tin, thallium and yttrium were in WTiO_2_.

**Figure 2:**
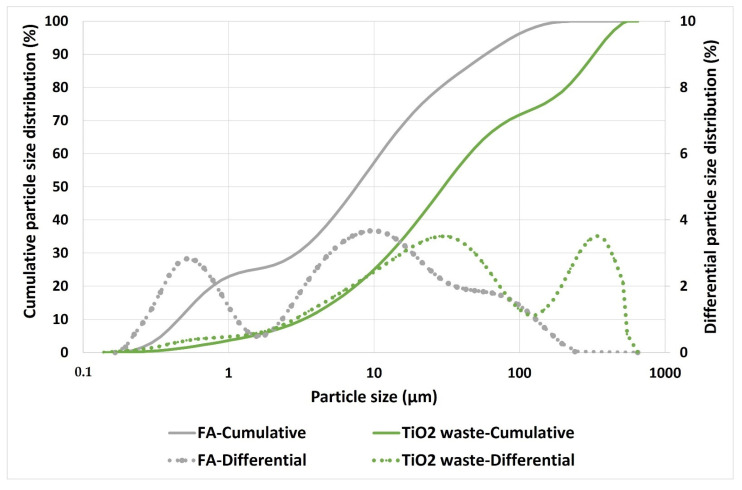
Particle size distribution of FA and TiO_2_ waste.

The particle size distribution of both materials is shown in Figure 2. The cumulative percentage is represented by continuous lines while the differential percentage is indicated by dotted lines. The D_50_ of FA and TiO_2_ waste was 7.59 and 30.08 µm, respectively.

The activating solution used in the geopolymerization process consists of a mixture of sodium silicate solution (NaSil) and sodium hydroxide pellets (NaOH (purity ≥ 98%) to reach a Na_2_O/SiO_2_ molar ratio of 0.98. 

### 2.2. Mix Design and Preparation of Samples

Compositions of the geopolymer samples prepared are detailed in Table 3, where the influence of replacing FA with TiO_2_ waste was analyzed. 

All components were mixed using a laboratory kneader. Firstly, solid precursors were poured into the kneader to mix them. After that, the activator solution was poured into the solid and all materials mixed until a homogeneous and thixotropic paste was formed. The next step was to fill cylindrical (diameter 3 cm-height 4 cm) molds and vibrate for 3 min to eliminate any internal bubbles. They were removed from the mold after 24 h at room temperature. The samples were left to cure for a further 27 days under room temperature conditions (25 °C and 65% of humidity). Samples were covered with cling film to avoid the carbonation process produced during exposition to ambient conditions [18].

### 2.3. Physical Properties

The sample bulk density has been obtained as an average result between three parallelepipeds weight and volume measurements using the EN 12390-7 standard method [19]. The humidity content (H) and water absorption capacity (A) were determined in three cylindrical samples, as specified in EN-12859 [20]. The degree of reaction was also determined in geopolymer with a FA/TiO_2_ waste ratio of 100/0 and 60/40. This measure was estimated as the percentage of soluble material after a three-hour attack of the samples with a hydrochloric acid dissolution. The reaction intensity provides some indication of the fly ash turned into geopolymers [21].

### 2.4. Thermogravimetric Analysis (TG)

Geopolymer samples with a weight between 100 and 150 mg were subjected to thermogravimetric analysis for the TG-SDTA measurements from 25 to 1000 °C with a heating rate of 20 °C per minute, using air as the purging gas [22].

### 2.5. Mechanical Properties

Once the sample was manufactured, mechanical characteristics were experimentally determined. To obtain representative values, the following tests were carried out using three specimens in each one. Superficial hardness was measured after 28 days of curing in accordance with EN-12859 [20]. A Shore C durometer was necessary to perform this test. At least 12 measurements were made for each sample on different faces. Surface hardness is the arithmetic average of at least six measurements expressed in Shore C units. Values with a dispersion greater than 10% were discarded to obtain an appropriate average.

Compressive strength was measured in accordance with ASTM-E-761-81 [23] at 7, 14 and 28 days after their manufacture, so that the evolution of resistance with curing time could be studied. In addition, the evolution of compressive strength after exposition to high temperatures (100, 300, 500 and 700 °C) for 3 h was evaluated. For this test, the resistance index (RI_T_) was ascertained, which is determined as the resistance coefficient at a temperature T, as:

RI_T_ (%) = (100·R_T_)/R_0_ (1)

R_0_ being the compressive value at 28 days (room temperature) and R_T_ the compressive value after exposition to high temperatures. Three samples were tested for each composition in both tests. 

### 2.6. Fire Insulating Capacity

Fire insulating capacity is defined as the time required to reach a temperature of 600 °C (T_600_) in the center of the sample (T_in_). Cylinder-shaped samples were baked and exposed to an external temperature in accordance with European standards [24] and as carried out in previous works [22,25]. Furthermore, ceramic fibers were used to isolate the top and bottom surfaces of the samples. Therefore, samples were hit with a symmetric radial heat flow. The fire temperature (T, °C) and the exposition time (t, minutes) are related as follows: T = 20 + 345·log(t + 1). This test was carried out after 28 days of curing. Larger cylinder molds were necessary (height 20 cm and diameter 4.2 cm) with a 10-cm wire inside. This wire must be as centered as possible, since it is where the thermocouple must be placed to measure the internal temperature of the sample when it is put into the oven, and it plays the role of the steel structure. 

### 2.7. Leaching and Radionuclide Activity Test 

The mission of hazardous substances was evaluated (European Landfill Directive [26]), and the EN-12457-4:2003 [27] leaching test was carried out. This leaching test offers information on granular waste and sludge behavior under experimental settings, specifically a liquid-to-solid ratio of 10 L/kg dry matter. It applies to material with particle sizes smaller than 10 mm.

In addition, due to the nature of TiO_2_ waste, a radionuclide activity test, consisting of determining of the 226-Ra, Th-232 and K-40 activities, was carried out. Two samples of each geopolymer were analyzed. The 226-Ra activity was determined through 214-Pb gamma emission, 40-K activity concentration was obtained from its 1460 keV gamma emission, and 232-Th from 228-Ac gamma emissions. A Canberra low-background high-purity germanium (HPGe) GR-6022 reverse electrode coaxial detector was used as the primary gamma-ray detector. It had a relative efficiency of 60% and was encased in a 10-cm thick high-purity lead shield [28].

## 3. Results

### 3.1. Physical Properties Results

Table 4 shows the physical properties results. Physical properties depend on the particle size distribution and specific gravity of raw materials. As can be seen in Figure 2, FA showed a lower particle size distribution, so higher packaging in the geopolymer should be produced and a higher bulk density was expected. However, as previously mentioned, TiO_2_ waste presents a higher specific gravity than FA. Therefore, although both opposite effects occurred, Table 4 shows that the effect of the specific gravity was somewhat more relevant, and the bulk density slightly increased when the TiO_2_ waste content was higher. Another parameter to take into account to explain physical properties in geopolymer matrixes is the degree of reaction, which has been calculated in geopolymer FA/TiO_2_ waste 100/0 and 60/40. Geopolymer 100/0 presented a degree of reaction of 52%, and the geopolymer 60/40 showed a value of 28% (slightly less than the theorical value of 31, corresponding to the 60%). As can be seen, TiO_2_ waste acts as an inert component in geopolymerization reaction and shows an inhibitory behavior which hinders the interaction between fly ash and the activating solution. Therefore, the greater the degree of reaction, the lower the porosity and higher the bulk density, as concluded in other works [21]. 

On the other hand, humidity content and water absorption capacity became lower with a higher amount of TiO_2_ waste, proving that they are inversely proportional to the bulk density [29]. 

### 3.2. Thermogravimetric Analysis

**Figure 3:**
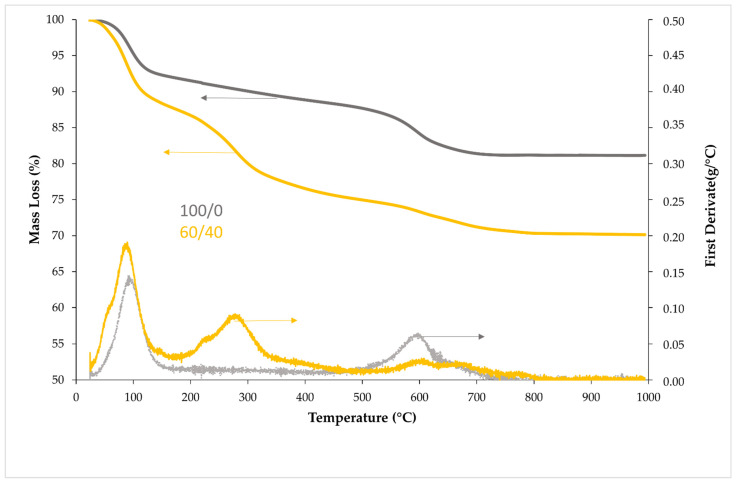
Thermogravimetric results.

Thermogravimetric results are shown in Figure 3 (left arrows-Mass Loss and right arrows-First Derivate). As can be seen, several mass losses corresponding to different endothermic reactions can be observed in 100/0 and 60/40 compositions. A first peak between 40 a 146 °C can be observed due to several reactions: (1) the evaporation of free and chemically bound water, (2) the decomposition of the C-S-H phase in both compositions and (3) the addition of TiO_2_ waste produced an increase in the sulfate content which reacts with Ca, producing Ca_2_SO_4_·2H_2_O and an increase in the mass loss in this range. All these peaks are overlapping due to the high heating rate used [30].

In the range of 200–350 °C, a second endothermic peak can be observed, but only in 60/40. This effect is possibly produced by the dehydration of Friedel salts (Ca_4_Al_2_O_6_Cl_2_∙10H_2_O). The XRD of TiO_2_ waste was carried out, and Friedel salts were visualized at 11.6° [31].

The third peak at 600 °C mainly corresponds to the endothermic decomposition of CaCO_3_ formed during the curing time, which is higher in 100/0 than 60/40. To avoid the carbonation process in geopolymers, samples were covered with cling film, but a certain level of carbonation is always produced. This process is more evident in geopolymers with a greater amount of fly ash, where the Ca content is higher. 

### 3.3. Mechanical Properties

#### 3.3.1. Superficial Hardness

**Figure 4:**
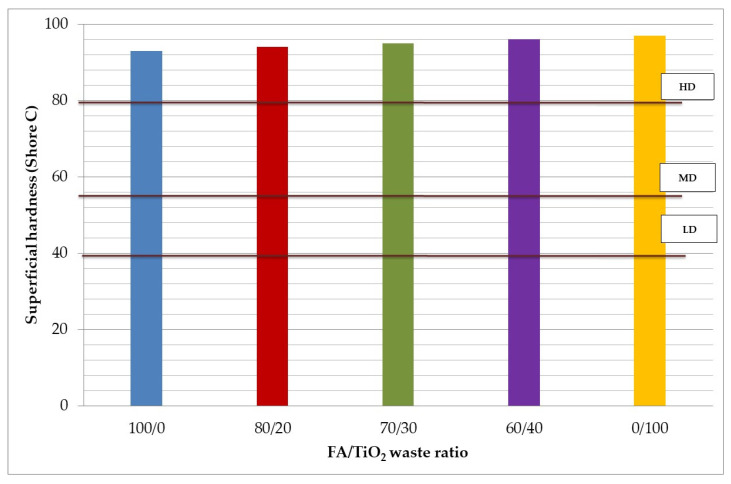
Superficial hardness results. Comparison with EN 12859.

Figure 4 presents the values of Superficial Hardness. All values were between 92 and 97 Shore C. Superficial hardness slightly increases as the FA/TiO_2_ waste ratio decreases, showing the same evolution as the bulk density; therefore, the final geopolymer resulted in a greater-hardness material. 

This method is related to the resistance with which the material opposes to the penetration of a normalized steel point. EN-12859 establishes 80 (expressed in Shore C units) as the high bulk density (HD) (>1100 kg/m^3^), 55 as medium (MD) (800–1100 kg/m^3^) and 40 as low (LD) (<800 kg/m^3^) for certain construction materials. As shown in Table 4, all the geopolymers studied can be classified as high bulk density, all of them satisfying the 80 Shore C (see Figure 4).

#### 3.3.2. Compressive Strength

**Figure 5:**
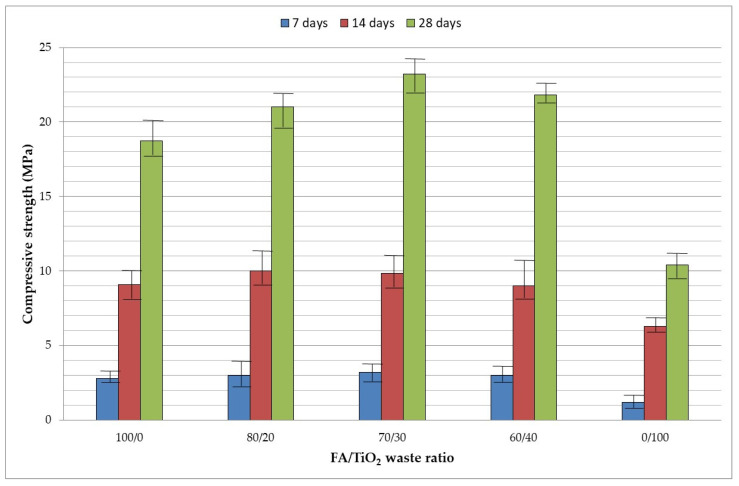
Compressive strength results.

As stated above, compressive strength (CS) was determined after 7, 14 and 28 days of curing at room temperature. Figure 5 shows the results of each group of geopolymers. CS at 7, 14 and 28 days increased as the TiO_2_ waste content rose to 30%. From 30%, CS began to lessen. A high dosage of the TiO_2_ waste increased the CS, possibly due to an increase in rigidity in the core because of the rutile (TiO_2_) [32], which is the main constituent (52.92%), as can be seen in Table 1. Rutile does not react, causing an amorphous structure to remain trapped in the matrix as an inert component providing rigidity to the core [33], reducing the mechanical properties when it is added in a high proportion (>30%). When the replacement of FA by TiO_2_ waste was higher, the compressive strength diminished due to obstruction of the geopolymerization process and reduced the compressive strength because TiO_2_ waste does not provide enough Si and Al.

### 3.4. Fire Insulating Properties 

**Figure 6:**
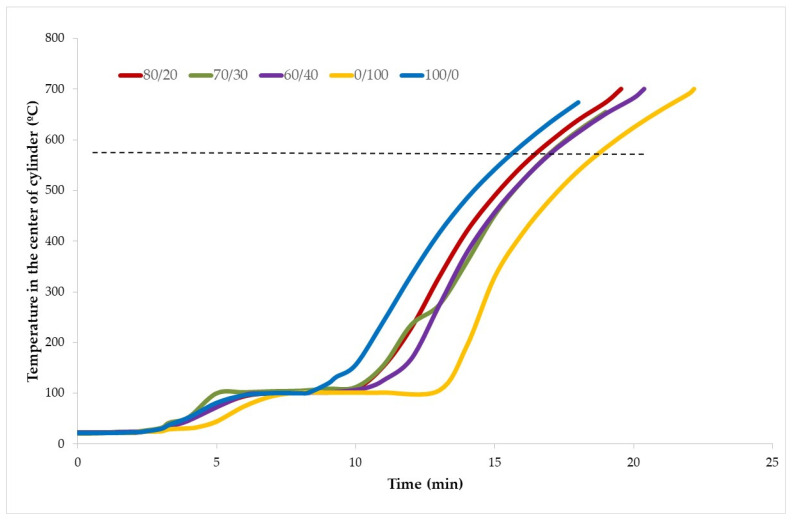
Fire resistance results.

Figure 6 shows the curve obtained after performing the fire resistance test. It is observed that the specimens had a similar slope in their initial section (between ambient temperature and around 100 °C). In addition, they all presented an evaporation plateau (period in which the temperature remains constant due to the evaporation of the water in the material) at around 100 °C, although not for the same duration. After the evaporation plateau, the slope of the curves is very similar for all the compositions.

As can be seen, when TiO_2_ waste was increased, fire resistance also declined because of the longer duration of the evaporation plateau due to the endothermic reactions produced (Figure 4), which absorb part of the energy of fire, maintaining a constant temperature in the center of the cylinder for a longer time [15]. 

Finally, it should be noted that no smoke or toxic gases were emitted during the test; only the water evaporated during the different endothermic reactions.

In addition, samples maintained their shape despite the cracks that appeared throughout.

**Figure 7:**
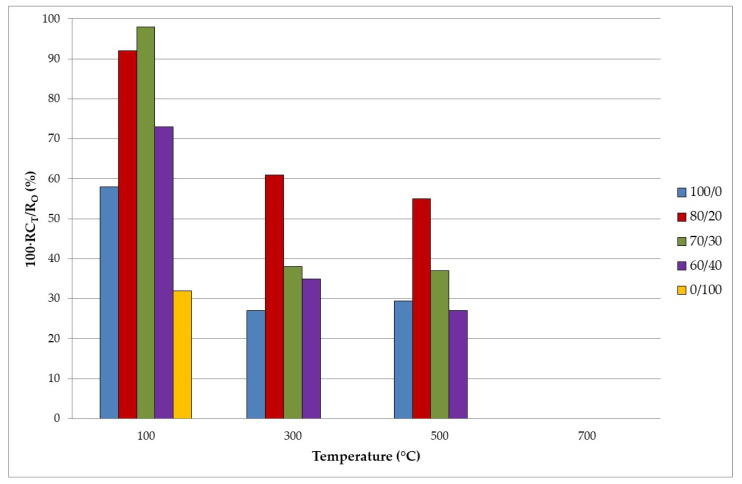
Evolution of compressive strength with temperature.

Figure 7 represents a comparison of the compressive strength of the samples at room temperature (RC_0_) after being exposed to different high temperatures (RC_T_). A drop in CS as the temperature increased can be observed due to the loss of mass and the chemical transformations that took place in the material during exposure to high temperatures (detailed in Section 3.2, Figure 3). This situation caused an increase in internal porosity and, therefore, in the development of breakage preferential pathways, leading to a decrease in compressive strength. Even for geopolymer 0/100 (without FA) at 300 °C, the mass loss that occurred was so great that the samples suffered a spalling process. The great mass loss obtained for all the samples at 700 °C produced a severe spalling, and there are no data at that temperature.

### 3.5. Leaching and Radionuclide Activity Test (Environmental Assessment)

Building materials must not pose a hazard to the safety, health or hygiene of people and must not have a high impact on the environment during their service life. According to the European Landfill Directive (EULFD) [25], three waste categories are defined: hazardous (HW), non-hazardous (N-H) and inert (I) wastes. Table 5 compares leaching data from the two wastes and geopolymers FA/TiO_2_ waste 100/0 and 60/40. Mo, Cr and Se concentrations in FA leachate were above the I limit, so the FA studied in this work can be considered a N-H waste. TiO_2_ waste presented an acidic pH (3.69). Zn, Pb, Cr and Ni concentrations were quite high (even the Cu concentration was over the H limits), so TiO_2_ waste must be treated before disposal in a landfill. Both geopolymers show high alkaline pH (close to 13). Low Ni, Zn, Cr, Pb, Cd, Ba, Cu and Se concentrations can be observed in both geopolymers, which demonstrate the effective immobilization of these metals. It is important to highlight the arsenic concentration in the leachates, 13 and 40 mg/kg in geopolymers 100/0 and 60/40, respectively, possibly due to the high pH value in the leachates. Arsenic solubility in soils is low at neutral or slightly acidic conditions, but the leachability is very significant in acidic and alkaline conditions [34].

Natural construction materials and wastes include radionuclides. The long-term exposure limit value for gamma radiation is set at 1.0 mSv/y by 2013/59/EURATOM Directive [35]. As can be seen in Equation (2), performance of a building material can be evaluated by calculating the activity concentration index (ACI), in which activity concentrations of the main natural radionuclides (such as Th-232, Ra-226 and K-40) are involved.

ACI = (CTh/200) + (CRa/300) + (CK/3000)(2)

where CTh, CRa and CK are, respectively, the activity concentrations (Bq/kg) of Th-232, Ra-226 and K-40. The ACI value ought to be under 1.0 [36] to accomplish the annual limit of 1.0 mSv/y. Table 6 shows the material activity concentrations and ACI values for geopolymers with a FA/TiO_2_ waste ratio of 100/0 and 60/40. As can be seen, the ACI was lower than 1 in both cases, although when the TiO_2_ waste was increased, Ra-226 and Th-232 concentrations increased and Ra-226 diminished, but ACI rose. Concrete concentrations are typically 260 Bq/kg (Ra-226), 190 Bq/kg (Th-232) and 1600 Br/kg (K-40), which are lower than the geopolymers studied in this work [37].

## 4. Conclusions

Based on the results of this work, the following conclusions can be drawn:

- The main goal has been achieved, since a mixture of TiO_2_ waste and FA can be used to manufacture geopolymers.
- From a physical properties point of view, the substitution of FA with TiO_2_ waste increases the bulk density due to its higher specific bulk density
- From a mechanical point of view, around 30–40% (*w*/*w*) of TiO_2_ waste reaches the highest compressive strength value. Compressive strength decreases when geopolymers are subjected to high temperatures, especially when more TiO_2_ waste is added.
- From a fire resistance point of view, when the amount of TiO_2_ waste increases, so does the plateau of evaporation, and this, in turn, increases the resistance to fire.
- From a leaching point of view, geopolymers produced a stabilization process of the heavy metals present in FA and TiO_2_ waste. Arsenic leaching was very important, possibly due to the high alkaline pH of leachates.
- From a radiological point of view, according to the European Directive on radiation in building materials, since ACI values are below 1, they could be used without harming people’s health; however, when TiO_2_ waste is increased, the ACI is increased.

## Figures and Tables

**Table 1:** Main components.

Component (%w)	FA	TiO_2_ Waste
Al_2_O_3_	21.31	2.37
BaO	0.18	0.09
CaO	3.01	0.65
Cl_2_O_7_	0.14	0.06
Cr_2_O_3_	0.03	0.57
CuO	0.03	0.06
Fe_2_O_3_	10.06	16.15
K_2_O	3.01	0.70
MgO	1.55	0.22
MnO	0.14	0.77
Na_2_O	1.04	0.35
Nb_2_O_5_	0.02	0.15
P_2_O_5_	0.29	0.03
PbO_2_	0.01	0.11
SO_3_	0.90	6.01
SiO_2_	53.77	17.66
SrO	0.07	0.01
TiO_2_	1.23	52.92
ZnO	0.06	0.03
ZrO_2_	0.04	0.97
Moisture 105 °C	0.05	3.71
LOI 750 °C	3.32	10.9
Specific Gravity (g/cm^3^)	2.17	2.83

**Table 2:** Minor components.

Component (mg/kg)	FA	TiO_2_ Waste
As	-	104
Co	98	-
Ga	100	-
Ge	102	-
Hf	-	96
Mo	98	-
Ni	101	-
Rb	213	-
Se	114	-
Sn	-	203
Ta	-	195
Y	-	184

**Table 3:** Mix design of geopolymers.

Geopolymer	FA (%w)	TiO_2_ Waste (%w)	Activating Solution (g)/FA (g)	H_2_0 (g)/TiO_2_ Waste (g)
100/0	100	0	0.77	-
80/20	80	20	0.78	-
70/30	70	30	0.79	-
60/40	60	40	0.81	-
0/100	-	100	-	0.81

**Table 4:** Physical properties results.

Geopolymer	Bulk Density (kg/m^3^)	Humidity Content (%w)	Water Absorption Capacity (%)
100/0	1638 ± 5	13.8 ± 0.7	11.9 ± 0.9
80/20	1670 ± 5	13.4 ± 0.6	10.8 ± 0.7
70/30	1686 ± 5	13.4 ± 0.8	10.6 ± 0.8
60/40	1708 ± 5	13.1 ± 0.5	10.5 ± 0.7
0/100	1774 ± 5	12.0 ± 0.5	9.8 ± 0.7

**Table 5:** EN 12457-4 leachability values and limits of different standards (mg/kg, dry base).

	pH	mg/kg									
		As	Mo	Zn	Pb	Cr	Ni	Cu	Ba	Cd	Se
FA	10.51	≤0.5	10	≤0,01	≤0,5	5.27	≤0.1	≤0.3	3.74	≤0.05	1.88
WTiO_2_	3.69	≤0.25	<0.25	48.8	0.78	17.6	9.97	151	0.32	0.06	0.44
100-0	12.74	13	2.26	<0.50	<0.25	0.32	0.19	0.29	0.12	<0.05	0.23
60-40	12.84	40	5.38	<0.50	<0.30	0,19	0.24	0.89	<0.10	<0.05	0.47
I		0.5	0.5	4	0.5	0.5	0.4	2	20	0.04	0.1
N-H		2	10	50	10	10	10	50	100	1	1
H		25	30	200	50	70	40	100	300	5	7

**Table 6:** Materials activity concentrations and ACI values.

	Materials Activity Concentrations and ACI Values	
Radionuclides	100/0	60/40
Ra-226 (Bq/kg)	21.8 ± 1.6	65.2 ± 2.1
Th-232 (Bq/kg)	0 ± 1.7	75.90 ± 2.9
K-40 (Bq/kg)	426.0 ± 6.9	286 ± 3.2
ACI	0.21	0.69

## Data Availability

Not applicable.

## References

[1] Davidovits J. (1991). Geopolymers: Inorganic polymeric new materials. J. Therm. Anal..

[2] Luhar I., Luhar S., Abdullah M.M.A.B., Razak R.A., Vizureanu P., Sandu A.V., Matasaru P.-D. (2021). A State-of-the-Art Review on Innovative Geopolymer Composites Designed for Water and Wastewater Treatment. Materials.

[3] Łach M., Pławecka K., Bąk A., Adamczyk M., Bazan P., Kozub B., Korniejenko K., Lin W.-T. (2021). Review of Solutions for the Use of Phase Change Materials in Geopolymers. Materials.

[4] Leiva C., Luna-Galiano Y., Arenas C., Alonso-Fariñas B., Fernández-Pereira C. (2019). A porous geopolymer based on aluminum-waste with acoustic properties. Waste Manag..

[5] Luna-Galiano Y., Leiva C., Arenas C., Arroyo F., Vilches L.F., Pereira C.F., Villegas R. (2017). Behavior of Fly Ash-Based Geopolymer Panels Under Fire. Waste Biomass Valorisat..

[6] Duxson P., Mallicoat S.W., Lukey G.C., Kriven W.M., Van Deventer J.S.J. (2007). The effect of alkali and Si/Al ratio on the development of mechanical properties of metakaolin-based geopolymers. Colloids Surfaces A Physicochem. Eng. Asp..

[7] Sofi M., Van Deventer J.S.J., Mendis P.A., Lukey G.C. (2007). Engineering properties of inorganic polymer concretes (IPCs). Cem. Concr. Res..

[8] Duxson P., Lukey G.C., Van Deventer J.S.J. (2006). Thermal conductivity of metakaolin geopolymers used as a first approximation for determining gel interconnectivity. Ind. Eng. Chem. Res..

[9] Palomo A., Grutzeck M.W., Blanco-Varela M.T. (1999). Alkali-activated fly ashes. A cement for the future. Cem. Concr. Res..

[10] Duxson P., Fernandez-Jimenez A., Provis J.L., Lukey G.C., Palomo A., Van Deventer J.S.J. (2007). Geopolymer technology: The current state of the art. J. Mater. Sci..

[11] Barbhuiya S., Pang E. (2022). Strength and Microstructure of Geopolymer Based on Fly Ash and Metakaolin. Materials.

[12] https://ec.europa.eu/eurostat/web/products-eurostat-news/-/ddn-20210810-1.

[13] Vilches L.F., Fernández-Pereira C., del Valle J.O., Vale J. (2003). Recycling potential of coal fly ash and titanium waste as new fireproof products. Chem. Eng. J..

[14] Li J., Wang W., Xu D., Wang X., Mao Y. (2020). Preparation of sulfoaluminate cementitious material using harmful titanium gypsum: Material properties and heavy metal immobilization characteristics. Waste Dispos. Sustain. Energy.

[15] Leiva C., Gómez-Barea A., Vilches L.F., Ollero P., Vale J., Fernández-Pereira C. (2007). Use of biomass gasification fly ash in lightweight plasterboard. Energy. Fuels.

[16] Vilches L.F., Leiva C., Olivares J., Vale J., Fernández C. (2005). Coal fly ash-containing sprayed mortar for passive fire protection of steel sections. Mater. Construcc..

[17] Gázquez M.J., Bolívar J.P., García-Tenorio R., Vaca F. (2009). Physicochemical characterization of raw materials and co-products from the titanium dioxide industry. J. Hazard. Mater..

[18] Luna-Galiano Y., Fernández Pereira C., Vale J. (2010). Waste Stabilization/Solidification (S/S) of EAF dust using fly ash-based geopolymers. Influence of carbonation on the stabilized solids. Coal Combust. Gasif. Prod..

[19] (2021). Testing hardened concrete—Part 7: Bulk Density of Hardened Concrete.

[20] (2012). Gypsum Blocks—Definitions, Requirements and Test Methods.

[21] Luna-Galiano Y., Fernández-Pereira C., Izquierdo M. (2016). Contributions to the study of porosity in fly ash-based geopolymers. Relationship between degree of reaction, porosity and compressive strength. Mater. Construct..

[22] Leiva C., Vilches L.F., Vale J., Fernández-Pereira C. (2005). Influence of the type of ash on the fire resistance characteristics of ash-enriched mortars. Fuel.

[23] (1991). Compressive Strength of the Fire-Resistive Material Applied to Structural Member.

[24] (2015). Fire Resistance Test. Part 1: General Requirements.

[25] Vilches L.F., Leiva C., Vale J., Fernández-Pereira C. (2005). Insulating capacity of fly ash pastes used for passive protection against fire. Cem. Concr. Compos..

[26] Council Directive 1999/31/EC of 26 April 1999 on the Landfill of Waste. Official Journal L 182, 16/07/1999 P. 0001–0019. European Commission (1999). http://data.europa.eu/eli/dir/1999/31/oj.

[27] (2003). Characterisation of Waste—Leaching—Compliance Test for Leaching of Granular Waste Materials and Sludges—Part 4: One Stage Batch Test at a Liquid to Solid Ratio of 10 l/kg for Materials with Particle Size below 10 mm.

[28] Leiva C., Arenas C., Cifuentes H., Vilches L.F., Rios J.D. (2017). Radiological, leaching, and mechanical properties of co-combustion fly ash in cements. J. Hazard. Toxic. Radioact. Waste.

[29] Luna-Galiano Y., Cornejo A., Leiva C., Vilches L.F., Fernández-Pereira C. (2015). Properties of fly ash and metakaolín based geopolymer panels under fire resistance tests. Mater. Construcc..

[30] Chyliński F., Bobrowicz J., Łukowski P. (2020). Undissolved Ilmenite Mud from TiO2 Production—Waste or a Valuable Addition to Portland Cement Composites?. Materials.

[31] An Q., Pan H., Zhao Q., Du S., Wang D. (2022). Strength development and microstructure of recycled gypsum-soda residue-GGBS based geopolymer. Constr. Build. Mater..

[32] Davidovits J., Comrie D., Davidovits J., Orlinski J. Long term durability of hazardous toxic and nuclear waste disposal. Proceedings of the 1est International Conference on Geopolymers`88.

[33] Gazquez M.J. (2010). Caracterización y Valorización de Residuos Generados en la Industria de Producción del Dioxido de Titanio. Ph.D. Thesis.

[34] Gersztyn L., Karczewska A., Gałka M.B. (2013). Influence of pH on the solubility of arsenic in heavily contaminated soils. Environ. Prot. Nat. Resour..

[35] Council Directive 2013/59/Euratom of 5 December 2013 Laying down Basic Safety Standards for Protection against the Dangers Arising from Exposure to Ionising Radiation, and Repealing Directives 89/618/Euratom, 90/641/Euratom, 96/29/Euratom, 97/43/Euratom and 2003/122/Euratom. https://www.legislation.gov.uk/eudr/2013/59/contents.

[36] Leiva C., Rodriguez-Galán M., Arenas C., Alonso-Fariñas B., Peceño B. (2018). A mechanical, leaching and radiological assessment of fired bricks with a high content of fly ash. Ceram. Int..

[37] Mas J.L., Caro Ramírez J.R., Hurtado Bermúdez S., Leiva Fernández C.L. (2021). Assessment of natural radioactivity levels and radiation exposure in new building materials in Spain. Radiat. Prot. Dosim..

